# Erythrocyte Membrane Incorporation Modulates the Stability and Fusion-Associated Behavior of Biomimetic Liposomes

**DOI:** 10.1021/acs.langmuir.6c01982

**Published:** 2026-07-30

**Authors:** Lucas R. Sousa, Luciano V. Sá, Tacio G. Hayasaki, Leticia S. O. Freitas, Karin A. Riske, Sebastião A. Mendanha, Eliana M. Lima

**Affiliations:** † 67824 FarmaTec - Laboratory for RD&I in Pharmaceutical Nanotechnology and Drug Delivery Systems, Samambaia Technology Park, 74690-631 Goiânia, Goiás, Brazil; ‡ Institute of Physics, Federal University of Goiás, 74690-900 Goiânia, Goiás, Brazil; § School of Pharmacy, Federal University of Goiás, 74605-170 Goiânia, Goiás, Brazil; ∥ Department of Biophysics, Federal University of São Paulo, 04039-032 São Paulo, São Paulo, Brazil

## Abstract

This study investigates how membrane composition governs the stability and fusogenic behavior of biomimetic hybrid liposomes composed of phosphatidylcholine (PC) and red blood cell (RBC) membrane fragments. While prior work on RBC-derived hybrid systems has largely focused on biological performance metrics such as circulation time, cellular uptake, and immune evasion, the biophysical and thermodynamic consequences of membrane hybridization at the molecular level remain poorly explored. Here, we address this gap through a systematic, multitechnique physicochemical characterization of a series of RBC-hybrid liposome formulations spanning a range of lipid:protein ratios. A comprehensive physicochemical characterization was performed using electron spin resonance (ESR) to probe membrane dynamics, microdifferential scanning calorimetry (microDSC) to assess thermotropic behavior, and isothermal titration calorimetry (ITC) to examine membrane energetics, solubilization, and intervesicular interactions. Incorporation of RBC membrane fragments increased membrane rigidity, as indicated by ESR and microDSC, and was associated with enhanced resistance to Triton X-100-induced solubilization, a finding attributed to compositional heterogeneity and protein–lipid organization introduced by membrane hybridization. ITC-based interaction assays further showed that cationic hybrid liposomes exhibited stronger fusion-associated signatures than their conventional counterparts, indicating that RBC membrane incorporation significantly alters interfacial membrane behavior in ways that cannot be explained by lipid composition alone. Together, these results show that RBC membrane content modulates both the structural stability and interaction profile of liposomes, establishing a biophysical framework for understanding how biomembrane-derived components reshape vesicle behavior and providing insights and useful design parameters for the development of robust biomimetic lipid-based delivery systems.

## Graphical abstract

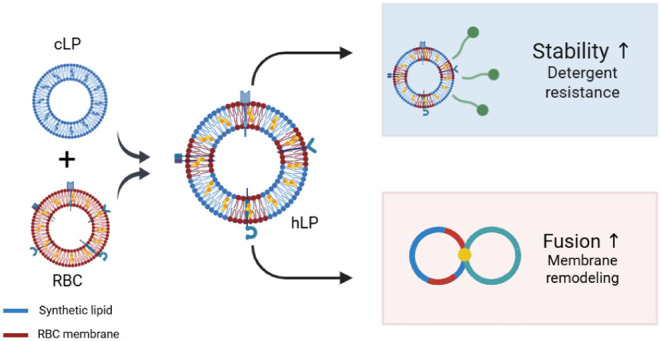

## Introduction

The development of efficient drug delivery systems capable of targeting specific tissues and cells remains a major challenge in nanomedicine.^1^ Diseases such as cancer, neurodegenerative disorders, and inflammatory conditions require therapeutic strategies that improve efficacy while minimizing systemic toxicity.^2–4^ In this context, liposomes have emerged as versatile nanocarriers because they can accommodate diverse therapeutic payloads and reshape pharmacokinetic behavior and effective drug exposure.^1,5,6^

Despite their broad potential, conventional liposomal systems still face important limitations. Instability in biological environments, immune recognition, and formulation-associated toxicity can compromise their clinical performance.^7–9^ Moreover, the membrane-level processes that govern their interactions with biological interfaces, including structural destabilization, particle internalization, and cargo release, remain incompletely understood. These limitations have driven increasing interest in biomimetic nanoparticles, which integrate biologically derived membrane components with synthetic carriers to better reproduce the complexity and functionality of natural membrane systems.^10,11^ Among these, lipid-based biomimetic platforms such as hybrid liposomes are particularly attractive because they retain the structural tunability of liposomes while incorporating compositional and biological features of cell-derived membranes.^11,12^

Within this class of biomimetic systems, red blood cell (RBC) membrane fragments are particularly appealing components. RBC-derived membranes possess a complex lipid–protein architecture and have been widely explored in biomimetic nanocarriers because of their favorable biocompatibility and association with prolonged circulation.^10,13^ When incorporated into liposomal formulations, these membrane components can substantially modify bilayer organization, rigidity, and interfacial behavior, with direct consequences for vesicle stability (membrane-level structural disruption) and membrane interaction pathways. Most studies on RBC-derived hybrid systems have, however, focused on biological performance metrics such as circulation time, cellular uptake, and immune evasion. How the incorporation of RBC membrane material alters membrane order, thermotropic behavior, detergent resistance, and intervesicular interaction energetics at the molecular level has received comparatively little attention, particularly across a systematic range of lipid:protein ratios.

A complete understanding of how membrane composition controls these properties is therefore essential. In this context, detergent-induced solubilization and membrane fusion are two particularly informative processes. Solubilization, often investigated using nonionic surfactants such as Triton X-100, involves a multistep transition from intact bilayers to mixed lipid-detergent assemblies and is governed by membrane packing, curvature stress, and detergent partitioning equilibria.^14,15^ Fusion, in turn, is a complex membrane-remodeling process that proceeds through intermediate states such as stalk formation, hemifusion, and pore opening, and is strongly influenced by lipid composition, membrane order, and bilayer organization.^16,17^ Together, these processes provide complementary insight into the structure–function relationships that govern membrane stability, disruption, and intervesicular interactions in lipid-based delivery systems.^18,19^

In this study, we investigate the detergent-induced destabilization and fusion-associated behavior of conventional and RBC-based hybrid liposomes, with emphasis on the physicochemical parameters that govern their structural integrity and interaction profile. Dynamic light scattering (DLS) and electrophoretic mobility measurements were used to evaluate vesicle size and zeta potential, respectively, whereas isothermal titration calorimetry (ITC), electron spin resonance (ESR) spectroscopy, and microdifferential scanning calorimetry (microDSC) were employed to characterize membrane energetics, order, fluidity, and thermotropic behavior. Through this integrated approach, we examine how incorporation of RBC membrane fragments modulates liposomal response to surfactant exposure and alters fusion-associated membrane behavior across a series of formulations spanning a defined range of lipid:protein ratios. These results are discussed in terms of the thermodynamic and spectroscopic evidence linking membrane composition to bilayer organization, detergent resistance, and interfacial interaction pathways, with the aim of establishing physicochemical design parameters for biomimetic vesicular systems.

## Materials and Methods

### Chemicals

Hydrogenated soybean phospholipids (HSPC, LIPOID P75–3) and highly unsaturated soybean phosphatidylcholine (SPC, LIPOID S100) were purchased from Lipoid GmbH (Ludwigshafen, Germany). Cholesterol (CHOL), 1,2-dioleoyl-3-trimethylammonium-propane (DOTAP), 1,2-dioleoyl-*sn*-glycero-3-phosphoglycerol (DOPG), 2-oleoyl-1-palmitoyl-*sn*-glycero-3-phosphocholine (POPC), the detergents t-octylphenoxypolyethoxyethanol (TX-100) and octyl β-d-glucopyranoside (OG), as well as the spin labels 5-doxyl-stearic acid (5-DSA) and 4-maleimido-1-oxyl-2,2,6,6-tetramethylpiperidine (6-MSL), were purchased from Merck (Darmstadt, Germany).

### Liposome Preparation

In this study, different liposomal formulations were employed. Conventional liposomes, denoted cLP, cLP_SPC_, and cLP_POPC_, were composed of HSPC, SPC, and POPC, respectively, each combined with CHOL at a 70:30 mol % ratio. The formulations cLP­(+) and cLP(−) consisted of an HSPC:CHOL base supplemented with cationic (DOTAP) or anionic (DOPG) lipids at a 60:30:10 mol % ratio. Hybrid liposomes (hLPs) were obtained by combining the HSPC:CHOL base with red blood cell membrane vesicles (cVS).

All conventional liposomes (total lipid concentration of 20 mM) were prepared by dissolving the lipids in chloroform, followed by thin-film formation by rotary evaporation under reduced pressure. The resulting lipid films were hydrated with ultrapure water to obtain multilamellar suspensions, which were then extruded at least 10 times through sequential polycarbonate membranes with pore sizes of 0.6, 0.4, 0.2, and 0.1 μm to produce unilamellar liposomes.

Hybrid vesicles were prepared from red blood cell membranes (mRBC) obtained by hypotonic lysis, as previously described.^20^ After removal of intracellular contents and membrane isolation, the membrane suspensions (∼5 mg protein/mL) were extruded 10 times through each of the same four polycarbonate membranes to generate unilamellar cVS. To produce hLPs, preformed conventional liposomes were mixed with RBC-derived membrane fragments at defined lipid:protein ratios (w/w) of 2:1, 4:1, 6:1, 8:1, and 10:1. The membrane fragments were added directly to the cLP suspension, and the resulting mixture was subjected to 20 freeze–thaw cycles followed by re-extrusion through the same pore-size sequence used for cVS preparation. This procedure was intended to promote membrane remodeling, lipid mixing, and coassembly of RBC-derived lipids and proteins with the synthetic bilayer, rather than producing a physical blend of the two components. Consequently, the total lipid content of the hybrid preparations comprises contributions from both the cLP and the membrane fragment fractions. A detailed overview of the lipid and protein contents of all formulations is provided in Table 1.

**Table 1:** Lipid Composition and Formulation Details of Conventional and Hybrid Liposomes

formulation	composition	lipid:protein (w/w)	protein (mg/mL)
cLP	HSPC:CHOL (70:30)	–	–
cLP_SPC_	SPC:CHOL (70:30)	–	–
cLP_POPC_	POPC:CHOL (70:30)	–	–
cLP(+)	HSPC:CHOL:DOTAP (60:30:10)	–	–
cLP(−)	HSPC:CHOL:DOPG (60:30:10)	–	–
hLP2:1	HSPC:CHOL (70:30) + mRBC	2:1	2.5 ± 0.2
hLP4:1	HSPC:CHOL (70:30) + mRBC	4:1	1.5 ± 0.1
hLP6:1	HSPC:CHOL (70:30) + mRBC	6:1	1.1 ± 0.1
hLP8:1	HSPC:CHOL (70:30) + mRBC	8:1	0.8 ± 0.1
hLP10:1	HSPC:CHOL (70:30) + mRBC	10:1	0.7 ± 0.1
hLP(+)	HSPC:CHOL:DOTAP (60:30:10) + mRBC	2:1	2.3 ± 0.2
cVS	mRBC	–	5.2 ± 0.2

### Liposome Characterization

Liposomes were characterized in terms of size, zeta potential, concentration, protein content, membrane dynamics, thermotropic behavior, and morphology. Particle size and zeta potential were measured using a Zetasizer Nano ZS90 (Malvern Panalytical). Particle concentration was determined by nanoparticle tracking analysis (NTA) using a NanoSight NS500 (Malvern Panalytical) equipped with a 532 nm laser and an EMCCD 215S camera.

Membrane dynamics were assessed by electron spin resonance (ESR) spectroscopy using an EMXPlus EPR spectrometer (Bruker) operating in the X-band (approximately 9.4 GHz). The instrumental parameters were as follows: microwave power, 2.0 mW; modulation frequency, 100 kHz; modulation amplitude, 1.0 G; magnetic field scan, 100 G; scan time, 168 s; and detection time constant, 41 ms. The maximum hyperfine splitting parameter (2A_||_), obtained directly from the experimental spectra, was used as an indicator of membrane rigidity, as previously described.^21^ All spectra were acquired at 25 °C.

The thermotropic behavior of the liposomal bilayers was analyzed using a MicroCal PEAQ-DSC instrument (Malvern Panalytical). Samples were degassed and loaded into the calorimetric cell, with ultrapure water used as reference. Scans were performed from 25 °C to 80 °C at a constant heating rate of 0.5 °C/min under a pressure of 50 psi. The differential power (DP) signal was recorded, and the thermograms were baseline-corrected using the manufacturer’s analysis software.

Protein incorporation into hybrid liposomes was first quantified using a BCA assay (Sigma-Aldrich) and further evaluated by SDS-polyacrylamide gel electrophoresis (SDS-PAGE). For SDS-PAGE analysis, samples were lysed, and membrane proteins were separated on 12% polyacrylamide gels under denaturing conditions, followed by Coomassie Brilliant Blue staining. This analysis enabled qualitative comparison of the protein profiles of hybrid liposomes, cell-derived vesicles, and native RBC membranes, confirming retention of representative membrane protein bands after extrusion and hybridization.

Finally, liposome morphology was evaluated by transmission electron microscopy (TEM) using a JEOL JEM-2100 microscope (Tokyo, Japan). Samples were fixed in buffered formaldehyde solution (2.5%, pH 7.0), postfixed with 4% osmium tetroxide, dehydrated in ethanol, deposited onto carbon-coated copper TEM grids, and stained with 0.5% aqueous uranyl acetate.

### Detergent Solubilization and Membrane Fusion Assays

Isothermal titration calorimetry (ITC) and ESR were used to evaluate membrane-detergent interactions. ITC experiments were performed using a PEAQ-ITC microcalorimeter (Malvern Panalytical). For solubilization assays, liposome suspensions and TX-100 or OG solutions were placed in the syringe and heat cell, respectively, at the desired concentrations for each experiment.

ESR spectra of the lipid- and protein-labeled fractions of hybrid liposome membranes after exposure to TX-100 were recorded as described above. All samples were labeled prior to detergent exposure. The lipid phase was labeled by incorporating 1 μL of 5-DSA spin probe (5 mg/mL in ethanol) directly into the lipid bilayer, whereas membrane proteins were labeled with 3 μL of 6-MSL spin probe (50 mg/mL in ethanol). Unbound 6-MSL was removed by centrifugation using Amicon Ultra 30 kDa filters.

Membrane interaction experiments were performed by titrating cationic liposomes, hLP­(+) or cLP­(+), at 10^12^ particles/mL from the syringe into a calorimetric cell containing 300 μL of an anionic liposome suspension at the same particle concentration.

### Heat Normalization

In this work, the heat variation Δ*Q* measured by ITC was assumed to predominantly reflect interfacial processes at the liposomal membrane, including detergent partitioning, surface interactions, lipid bilayer reorganization, and other events that scale with the available membrane surface area. Accordingly, normalization of the calorimetric signal by the total membrane area present in each sample was performed to enable direct comparison among systems differing in both particle size and particle number, thereby minimizing purely geometric bias. Because detergent partitioning and intervesicular interactions are initiated at the externally accessible membrane surface, only the outer leaflet area was considered for normalization.

Liposomes were approximated as spherical particles with average hydrodynamic diameter *D*, determined by DLS, and total particle number *N*, obtained by NTA. Under these assumptions, the surface area of an individual liposome is given by

1 $$A_{\mathrm{lipo}}=4\pi R^{2}=\pi D^{2}$$

where R = *D*/2 is the mean particle radius. The total membrane area in each sample, considering only the outer leaflet of the bilayer, is therefore

2 $$A_{\mathrm{tot}}=N\pi D^{2}$$

The experimentally measured heat variation Δ*Q* was normalized by the corresponding total membrane area, yielding an effective heat variation per unit area:

3 $$\Delta Q_{\mathrm{area}}=\frac{\Delta Q}{N\pi D^{2}}$$

## Results and Discussion

An overview of the liposomal systems investigated in this study is presented in Figure 1A, which schematically depicts conventional liposomes (cLP), cell-vesicle systems (cVS), and hybrid liposomes (hLP), highlighting the compositional and structural changes introduced by membrane hybridization. Hydrogenated soybean phosphatidylcholine (HSPC), containing approximately 70–75% saturated phosphatidylcholine, was used as the main lipid component for liposome preparation. Saturated phospholipids of this class are widely employed in liposomal formulations because of their high purity, biocompatibility, and ability to form mechanically robust bilayers.^22^ Their high acyl-chain saturation promotes tight lateral packing, reduced free volume in the hydrophobic core, and increased membrane rigidity, thereby contributing to enhanced thermodynamic and colloidal stability. In addition, the residual compositional heterogeneity of the extract, including minor fractions of other phospholipids and neutral lipids, may increase interfacial complexity and better approximate the physicochemical features of biological membranes.

**Figure 1:**
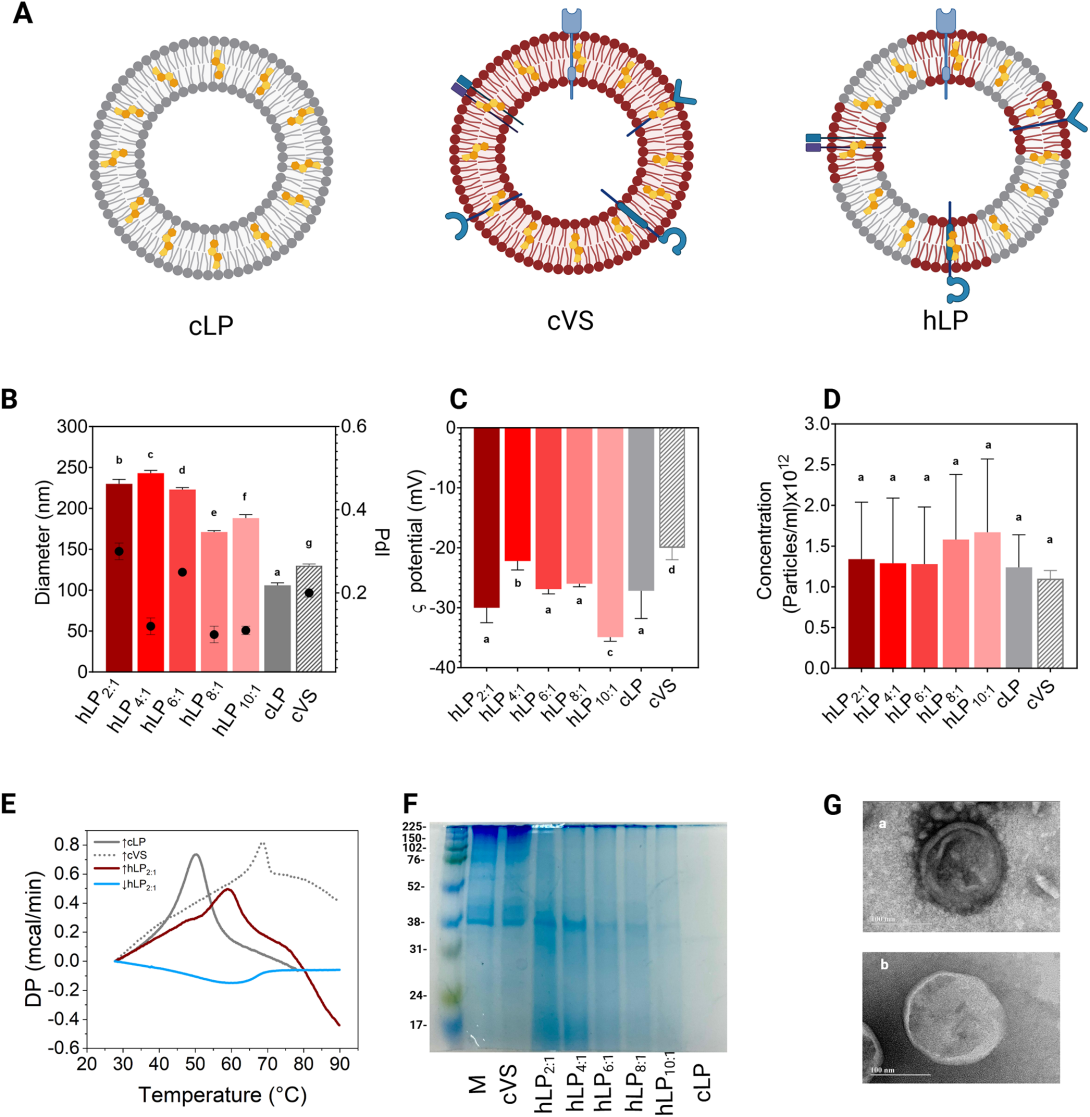
(A) Schematic representation of the liposomal systems investigated in this study, including conventional liposomes (cLP), cell-vesicle systems (cVS), and hybrid liposomes (hLP). (B) Mean hydrodynamic diameter of the vesicular formulations determined by DLS, together with their respective polydispersity index (PdI). (C) Zeta potential and (D) particle concentration of all formulations. Data in (B–D) are expressed as mean ± SD (*n* = 3). Different letters above bars indicate statistically significant differences among formulations (*p* < 0.05). (E) Thermotropic behavior assessed by microDSC, showing phase transition profiles associated with bilayer organization and hybrid membrane composition. Up-pointing arrows indicate heating scans, and down-pointing arrows indicate cooling scans. (F) SDS-PAGE profiles of RBC membrane fragments (M), cVS, and hybrid formulations, shown in comparison with protein-free cLP, demonstrating incorporation of erythrocyte membrane proteins after hybridization. (G) Representative transmission electron microscopy (TEM) micrographs of hybrid liposomes (a) and conventional liposomes (b).

As shown in Figure 1B, conventional liposomes displayed an average hydrodynamic diameter of approximately 100 nm, whereas all hybrid formulations exhibited larger diameters, ranging from approximately 175 to 250 nm. On average, hybrid vesicles were about 75 nm larger than cLP, consistent with incorporation of RBC-derived lipid and protein components into the bilayer. Similar size increases have been reported for RBC-derived hybrid liposomes, in which the synthetic lipid:erythrocyte membrane ratio influenced vesicle diameter, lamellar organization, and membrane packing.^12^ In this context, the increase in vesicle size observed here is consistent with structural remodeling of the membrane following hybridization.

Despite this increase in diameter, the PdI values remained below 0.4 for all formulations. Although the interpretation of PdI depends on the system under study, values below 0.3 are typically associated with highly uniform liposomal suspensions, whereas values between 0.3 and 0.5 are generally considered acceptable for experimental and preclinical formulations.^23^ Thus, the PdI values obtained here indicate that hybridization increased vesicle size without causing extensive heterogeneity or loss of preparation reproducibility. The maintenance of relatively narrow size distributions across different lipid:protein ratios further supports the robustness of the preparation protocol. In addition, the larger diameters observed for the hybrid systems are consistent with the use of saturated phosphatidylcholine-rich membranes, which are expected to favor more ordered bilayers and increased vesicle rigidity.^24^

The zeta potential values of the hybrid vesicles (Figure 1C) ranged from approximately −20 to −30 mV, in good agreement with previous reports for erythrocyte-derived membrane systems, which typically exhibit surface potentials around −25 mV.^12,25^ This similarity suggests that the surface properties of the hLPs partially preserve the electrostatic characteristics of native RBC membranes. Besides, conventional liposomes displayed zeta potential values within the same range as the hybrid formulations, consistent with previous observations for zwitterionic phosphatidylcholine-based membranes. Although electrically neutral in composition, such systems often exhibit modestly negative zeta potentials because of headgroup orientation, interfacial hydration, and ion adsorption effects. Previous studies have shown that HSPC-rich formulations tend to exhibit moderately negative surface potentials and increased rigidity relative to more unsaturated phosphatidylcholine systems.^26^ The absence of a clear compositional trend across the hLP series reflects the complex interplay between the lipid bilayer matrix and RBC-derived membrane components, including proteins, glycolipids, and phospholipids, each contributing differently and nonadditively to surface charge density. Despite statistically significant differences among select formulations, the narrow absolute variation observed suggests that surface electrostatics are governed primarily by the phosphatidylcholine-rich bilayer backbone, with RBC-derived components contributing only modestly regardless of the mixing ratio, and all formulations remained sufficiently negative to suggest moderate resistance to electrostatic aggregation, although a dedicated long-term colloidal stability study was not conducted in this work.

As shown in Figure 1D, all formulations exhibited particle concentrations on the order of 10^12^ particles/mL. Because hybrid liposomes were not prepared by simple mixing of cLP and cVS, but rather by coprocessing RBC-derived membrane fragments with cLP through freeze–thaw cycles followed by re-extrusion, particle concentration is not expected to scale additively with the input components. Instead, the re-extrusion step governs the final vesicle number by redistributing the total lipid and protein mass into a reformed particle population of larger mean size. The broadly similar concentrations observed across all hLP formulations (approximately 1.3–1.6 × 10^12^ particles/mL) indicate that incorporation of RBC membrane fragments did not substantially alter vesicle yield under the preparation conditions employed. The preservation of particle number across formulations is important because it supports comparison of physicochemical properties on a compositional basis rather than as a consequence of large differences in vesicle abundance. These results also indicate that the extrusion and hybridization procedures remained efficient even after inclusion of biologically derived membrane material.

The thermotropic behavior of the vesicular systems was examined by microDSC (Figure 1E), providing insight into bilayer packing, transition cooperativity, and membrane heterogeneity. Conventional liposomes displayed a well-defined endothermic transition characteristic of saturated phospholipid bilayers, consistent with the cooperative gel-to-liquid crystalline transition expected for HSPC-rich membranes. In contrast, hybrid formulations exhibited broader and, in some cases, shifted thermal events, indicating reduced cooperativity and increased lateral heterogeneity. These changes are consistent with incorporation of erythrocyte-derived lipids and proteins, which are expected to perturb the regular packing of saturated acyl chains and introduce domains with distinct thermodynamic behavior. The calorimetric profiles therefore support the view that membrane hybridization produces compositionally more complex bilayers with altered rigidity and phase behavior. To assess the reversibility of the observed transition in the hLP, an additional cooling scan was performed starting from the higher-temperature state under the same experimental conditions. The cooling thermogram did not recover the original endothermic transition profile; instead, an exothermic event was observed, indicating that the heating-induced structural reorganization of the hybrid membrane does not simply revert upon cooling. This behavior is consistent with irreversible or kinetically trapped rearrangements involving the lipid matrix and membrane-associated biomolecular components.

The electrophoretic profiles shown in Figure 1F provide qualitative evidence of membrane protein incorporation into the hybrid vesicles. As expected, conventional liposomes showed no detectable protein bands, confirming the absence of membrane-associated biomolecules in the purely synthetic formulation. In contrast, cVS displayed the complex band pattern characteristic of erythrocyte membrane material over a broad molecular weight range. Hybrid liposomes retained representative portions of this protein profile, although with lower band intensity than cVS, consistent with partial incorporation and dilution within the synthetic lipid matrix. These results support successful transfer of RBC-derived membrane proteins during the hybridization process and reinforce the biomimetic character of the hybrid vesicles.

Representative TEM images (Figure 1G and Figure S1) revealed predominantly rounded to quasi-spherical vesicles with preserved membrane contours, indicating maintenance of bilayer integrity after hybridization. Hybrid liposomes appeared larger than conventional liposomes, an observation qualitatively consistent with the DLS results, which provide the primary quantitative size distribution data. No extensive aggregation, rupture, or collapse was observed in the examined micrographs, suggesting that the hybridization procedure preserved vesicle integrity while promoting nanoscale structural remodeling. Together with the physicochemical and electrophoretic data, the TEM analysis supports the formation of structurally stable hybrid vesicles containing RBC-derived membrane components, providing complementary morphological evidence that the overall vesicular architecture is retained following hybridization.

Figure 2 shows the effects of Triton X-100 (TX-100) on the lipid and protein dynamics of cLP and hLP, as assessed by ESR spectroscopy. In Panels A and C, the spectra of the 5-DSA and 6-MSL spin labels were resolved into two components, corresponding to strongly immobilized (S) and weakly immobilized (W) populations. Because the 2A_||_ parameter is directly related to magnetic anisotropy and, therefore, to the motional restriction experienced by the spin label, it is more reliably determined from the S component.

**Figure 2:**
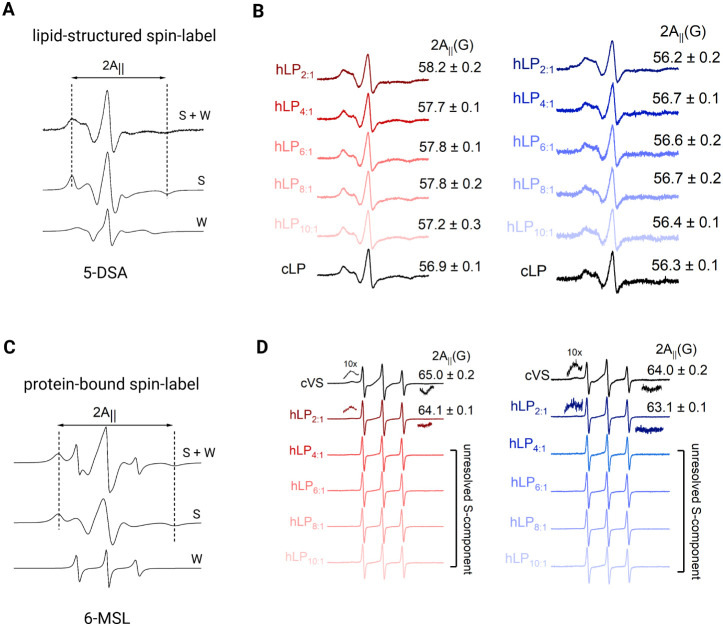
ESR spectra of spin-labeled liposome membranes before (red) and after (blue) exposure to TX-100 (0.01% v/v). Panels A and C illustrate the deconvolution of the experimental spectra into strongly immobilized (S) and weakly immobilized (W) components for each spin label. 5-DSA was incorporated into the lipid phase of the bilayer (panel B), whereas 6-MSL was covalently attached to SH groups of membrane proteins in hybrid liposomes (panel D). The 2A_||_ values obtained for 5-DSA are shown in panel B as mean ± SD. For 6-MSL, the outer hyperfine splitting of the S-component line used to calculate 2A_||_ is shown at 10× magnification for the spectra in which it could be resolved. Cell-derived vesicles (cVS) from RBC membranes were used as reference samples for 6-MSL labeling. Spectral intensities were normalized, and the magnetic field range was 100 G. Ratios from 2:1 to 10:1 denote the lipid:protein ratio (w/w) of each hybrid liposome formulation.

The spectra obtained with 5-DSA, which partitions into the lipid bilayer, indicate that incorporation of RBC membrane fragments alters membrane rigidity in all hybrid formulations. The 2A_||_ values obtained with 5-DSA were consistently higher in all hLP formulations compared to cLP (e.g., hLP_2:1_: 58.2 ± 0.2 G vs cLP: 56.9 ± 0.1 G), indicating increased lipid acyl-chain immobilization attributable to boundary lipid interactions with incorporated RBC membrane proteins. ESR spectra of fatty-acid spin labels in biological membranes are commonly interpreted as a superposition of a more mobile W component, associated with probes in the bulk lipid phase, and a more immobilized S component, associated with boundary lipids in the vicinity of membrane proteins. Although the relative contributions of the W and S components were not resolved quantitatively through spectral deconvolution, their relative prominence can be assessed from characteristic line shape features, following the qualitative framework adopted in previous ESR studies employing the same spin labels in erythrocyte membrane systems and lipid bilayers.^21,27–29^ Consistent with its higher protein content, hLP_2:1_ exhibited a spectrum whose line shape was dominated by the S component, as evidenced by the pronounced broadening of the outer hyperfine lines, indicating a larger contribution from lipid–protein interfacial regions. As the protein content decreased in the other formulations, the W component became progressively more evident, consistent with increased probe mobility.

Addition of TX-100 at concentrations below its critical micelle concentration (CMC), increased the fluidity of all vesicular systems, regardless of RBC membrane content. This effect is reflected by the reduction of up to 2 G in the 2A_||_ values and by the lower relative intensity of the S component. These results indicate that, even below the CMC, TX-100 partitions into the membrane and increases local dynamics without causing complete solubilization. The absence of a clear dependence on RBC content further suggests that, under these conditions, TX-100 primarily modulates interfacial lipid dynamics rather than inducing full membrane disruption.

Complementary information was obtained with the 6-MSL spin label, which is covalently attached to SH groups in membrane proteins and therefore reports on the mobility of protein-associated domains. For this label, the W component is characterized by three sharp isotropic lines, typical of nitroxide radicals in aqueous environments, whereas the S component reflects more restricted motion associated with protein-bound environments. Accordingly, the 2A_||_ values determined from the outer hyperfine splitting of the S component provide information on the segmental mobility of the labeled protein regions in the hybrid liposomes. A more detailed discussion of the interpretation of these spin labels and their spectral components can be found elsewhere.^21,27–29^

Analysis of the 6-MSL spectra showed that cVS exhibited higher 2A_||_ values than the hybrid liposomes, indicating more restricted motion of labeled protein-associated regions in the native erythrocyte membrane system. At higher lipid:protein ratios, such as 4:1, the S component became less distinct, which limited accurate determination of 2A_||_ in some cases.

Previous studies have shown that the spin-labeled protein fraction of RBC membranes produces composite ESR spectra with relatively low S-component intensity, suggesting that a substantial fraction of the reactive residues remains solvent-exposed.^21,29^ In this context, the progressive decrease in S-component intensity observed upon hybridization at higher lipid content suggests that the synthetic lipid environment alters protein-associated dynamics and may favor conformations with greater solvent exposure. Exposure to TX-100 decreased the 2A_||_ values of both cVS and hLP samples, consistent with detergent-induced changes in the local environment of membrane proteins. At the concentration used here, below the CMC, TX-100 partitions into the bilayer as monomers and preferentially associates with protein–lipid interfacial regions of locally altered acyl-chain packing, modulating local structure and mobility without fully disrupting the bilayer, consistent with established models of sub-CMC detergent-membrane interaction^30–33^ and supported by the observed changes in the ESR spectra and spectral parameters.

Detergent-resistant membranes (DRMs) are cholesterol- and saturated lipid-rich domains that resist solubilization by nonionic detergents such as TX-100 and are often associated with membrane organization and trafficking processes.^34^ Moreira et al. used 5-DSA and 6-MSL to evaluate the effects of an alkylphospholipid detergent on both the lipid and protein fractions of RBC membranes and their DRMs.^35^ They reported 2A_||_ values of 58.6 and 65.8 G for DRMs labeled with 5-DSA and 6-MSL, respectively. The 5-DSA value is close to that observed here for hLP_2:1_ (58.2 ± 0.2 G), suggesting that fusion of cholesterol-rich HSPC lipids with high fractions of erythrocyte membrane material produces a bilayer with lipid dynamics resembling those of DRM-like environments. In contrast, the slightly lower 6-MSL value observed in this study (64.1 ± 0.1 G) suggests that the labeled protein regions in hLPs remain somewhat more exposed to the aqueous environment than in native DRMs. Moreira et al. also reported marked increases in both lipid and protein dynamics after detergent treatment, consistent with the behavior observed here after TX-100 exposure.

The detergent-induced interaction dynamics of the hybrid liposomes were further investigated by isothermal titration calorimetry (ITC). Under submicellar conditions, TX-100 is predominantly present as monomers, which makes this regime suitable for probing the early stages of detergent-membrane interaction. In these experiments, TX-100 was placed in the calorimetric cell, and conventional or hybrid liposomes were titrated from the syringe, allowing detection of the heat changes associated with detergent partitioning into the membrane.

Under these conditions, the thermograms displayed relatively small heat changes that progressively decreased with successive injections (Figure 3A). This behavior is consistent with partitioning of monomeric TX-100 into the liposomal bilayer without full solubilization. The absence of a clear multiphasic thermogram, with distinct enthalpic transitions, is expected in the absence of micellar detergent and agrees with previous descriptions of detergent-lipid interactions below the critical micelle concentration (CMC).^30^ Thus, at the TX-100 concentration used here, the calorimetric response primarily reflects early interaction events, including detergent insertion into the bilayer and progressive membrane saturation, without transition to complete bilayer disruption or formation of homogeneous mixed micelles.

**Figure 3:**
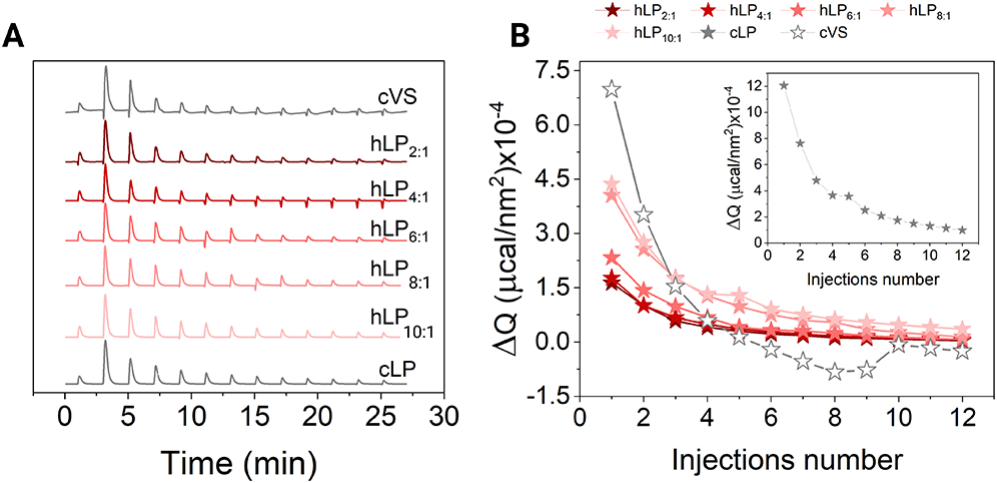
Isothermal titration calorimetric characterization of liposome interactions with Triton X-100 under submicellar conditions. (A) Representative thermograms obtained from titration of liposomes into TX-100. The syringe was filled with liposomes at a concentration of approximately 10^12^ particles/mL, whereas the cell contained 0.01% (v/v) TX-100 (165 μM). Titrations consisted of 13 injections of 3 μL (first injection: 0.4 μL). cVS, cLP, and hLP denote cell-derived vesicles, conventional liposomes, and hybrid liposomes, respectively. Ratios from 2:1 to 10:1 indicate the lipid:protein mass ratio (w/w) in the hLP formulations. (B) Heat variation per injection (Δ*Q*) derived from the ITC titrations. All measurements were performed at 25 °C.

Figure 3B shows the heat variation per injection during liposome titration into TX-100. For both conventional and hybrid liposomes, the endothermic signal (Δ*Q*) decreased monotonically with successive injections, indicating progressive saturation of the membrane with detergent. This trend further supports the conclusion that, although membrane perturbation occurs, complete solubilization is not reached under these conditions. Among the hybrid formulations, systems containing higher fractions of RBC membrane material exhibited a more sustained calorimetric response, whereas formulations with lower RBC content showed a faster reduction in Δ*Q*. Together with the ESR results, this behavior suggests that membrane composition modulates the extent of detergent interaction and that RBC-rich hybrid membranes are more resistant to detergent-induced perturbation.

These observations are consistent with established models of detergent-lipid interactions, in which the absence of micelles restricts the process to relatively mild membrane remodeling.^30,31^ In this context, the behavior of the hybrid liposomes indicates increased structural resilience under sub-CMC conditions. This effect is plausibly related to the compositional complexity of the biomimetic bilayer, particularly the presence of cholesterol-rich and protein-containing RBC membrane domains. RBC membranes are known to contain high cholesterol fractions, which contribute to membrane stabilization and preservation of protein function.^36^ Increased cholesterol content promotes tighter lipid packing and enhanced bilayer rigidity, in agreement with the higher membrane order indicated by ESR and the altered thermotropic behavior observed by microDSC. Cholesterol-rich membranes are also known to display reduced susceptibility to TX-100 solubilization, often undergoing only partial disruption even at higher detergent concentrations.^37^ Accordingly, the delayed response observed for RBC-rich hLP formulations is more consistent with partial membrane remodeling than with complete bilayer disintegration.

To further examine whether complete solubilization could be approached, additional ITC experiments were performed with TX-100 at supramicellar concentrations of 0.5, 0.75, and 1.0 mM using hLP_2:1_ (Figure 4). Under these conditions, the detergent is expected to be present predominantly as micelles, allowing access to later stages of detergent-membrane interaction, including membrane saturation and eventual bilayer disintegration into mixed micellar structures. However, even at these concentrations, the thermograms still showed a progressive decrease in endothermic heat with successive injections and, at later stages, the appearance of exothermic contributions.

This nonclassical calorimetric behavior suggests that hLP_2:1_ displays substantial resistance to TX-100-induced destabilization, which may delay or alter the transition to full solubilization. The high content of RBC-derived components, including cholesterol and membrane proteins, likely contributes to a tightly packed and compositionally heterogeneous bilayer that resists detergent-induced disassembly more strongly than simpler lipid systems.^30^ In this respect, the hybrid formulation displays a detergent-response profile that differs from that expected for more compositionally homogeneous liposomes.

The appearance of exothermic contributions after an initial sequence of diminishing endothermic events indicates a change in the dominant molecular processes occurring at the membrane interface. One possible explanation is that, as the bilayer approaches detergent saturation, further incorporation of TX-100 promotes local rearrangements that increase favorable lipid-detergent packing and release structured water, both of which can contribute exothermically.^30,33,38–40^ Another possibility is redistribution of detergent between bilayer-associated and aqueous states, reflecting dynamic equilibration processes that also alter the enthalpic signature.^30,32,33,40,41^

The hybrid liposomes, particularly hLP_2:1_, also introduce additional complexity because they contain membrane proteins. It is therefore plausible that part of the calorimetric response arises from detergent interaction with protein-associated regions of the membrane, including local protein-detergent or protein–lipid-detergent rearrangements.^42–44^ More generally, the emergence of exothermic signals may reflect the formation of transient intermediate states preceding full solubilization, such as detergent-enriched regions or metastable microdomains within the bilayer.^30,32,33,38,40^

**Figure 4:**
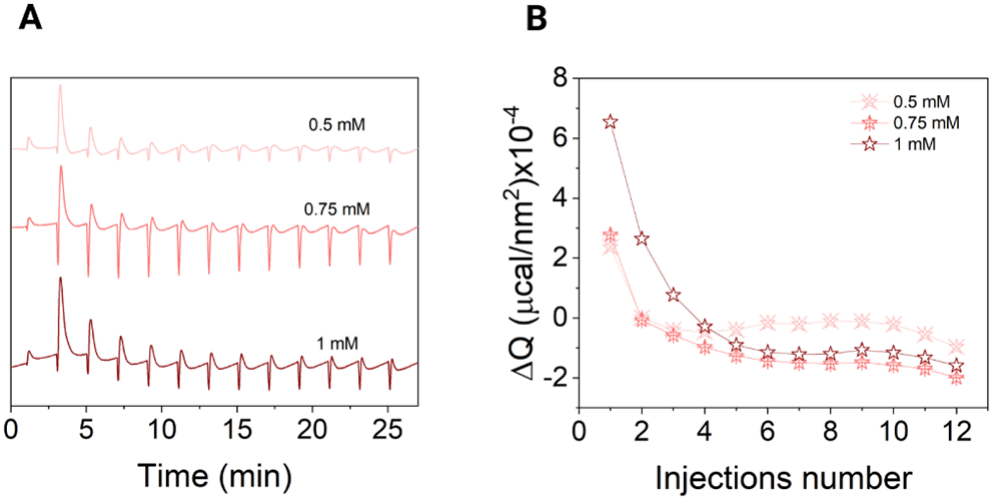
Thermograms obtained from titration of liposomes into TX-100. (A) The syringe was loaded with hLP_2:1_ at approximately 10^12^ particles/mL, and the cell was filled with TX-100 at supramicellar concentrations of 0.5, 0.75, and 1.0 mM. Each titration comprised 13 injections of 3 μL, with an initial injection of 0.4 μL. (B) Heat variation per injection (Δ*Q*) derived from the ITC titrations. All measurements were performed at 25 °C.

To further compare detergent-response profiles across different membrane compositions, we performed additional ITC experiments using octyl β-d-glucopyranoside (OG), a nonionic detergent commonly used in membrane solubilization studies. In these experiments, OG was used above its CMC in order to access the full solubilization regime.

For the conventional POPC:CHOL and SPC:CHOL liposomes, used here as compositional reference systems, the resulting thermograms followed the classical three-stage solubilization pattern (Figure 5), in agreement with previous descriptions of detergent-membrane interactions in simpler lipid systems. In contrast, HSPC:CHOL liposomes displayed an intermediate profile in which small exothermic contributions emerged after the initial endothermic regime, suggesting local structural rearrangements or formation of detergent-enriched membrane regions. These three conventional formulations were included to place the hybrid liposome behavior in the context of the classical solubilization framework, spanning a compositional range from the highly unsaturated POPC to the fully saturated HSPC, which already deviates from the classical profile at the intermediate stage.

**Figure 5:**
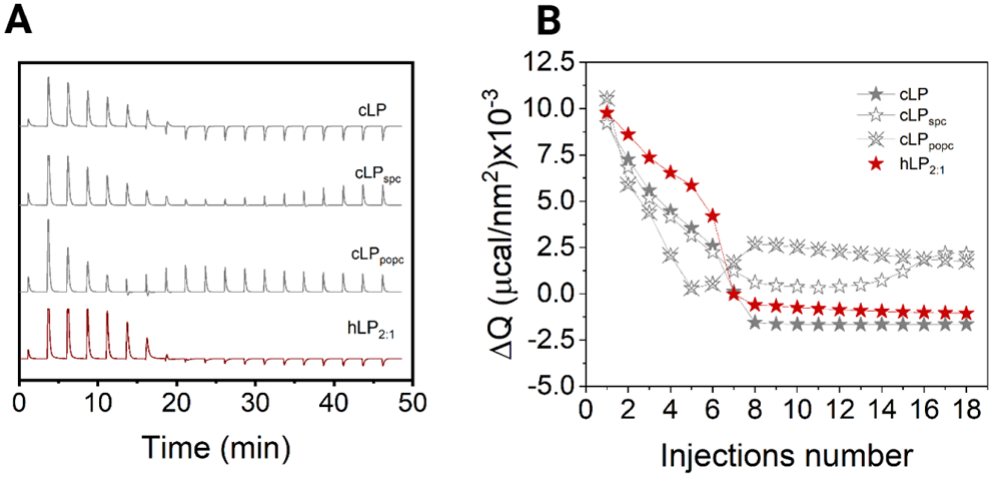
Isothermal titration calorimetric characterization of conventional and hybrid liposome interactions with octyl β-d-glucopyranoside under supramicellar conditions. (A) Representative thermograms obtained upon titration of liposomes into OG. The syringe was loaded with liposomes at approximately 10^12^ particles/mL, and the cell contained OG at a supramicellar concentration of 25 mM. Each titration comprised 13 injections of 3 μL, with an initial injection of 0.4 μL. cLP = HSPC:CHOL, cLP_SPC_ = SPC:CHOL, and cLP_POPC_ = POPC:CHOL. (B) Heat variation per injection (Δ*Q*) derived from the ITC titrations. All measurements were performed at 25 °C.

Hybrid liposomes displayed a distinct response. A progressive decline in endothermic heat was again observed with successive injections, indicating initial interaction of the detergent with the membrane and partial bilayer saturation. However, the absence of well-defined exothermic transitions suggests that the subsequent stages of membrane reorganization and mixed micelle formation were delayed or attenuated. This behavior is consistent with the greater structural integrity and compositional complexity imparted by the RBC-derived membrane components, which appear to hinder complete solubilization even in the presence of excess OG. Notably, hybrid liposomes with higher RBC content showed the most pronounced deviation from the classical profile, deviating most substantially from the behavior of all conventional references and indicating that biological membrane complexity progressively alters the solubilization pathway in a composition-dependent manner.

Beyond their role in modulating membrane stability and detergent response, bilayer structural properties are also central determinants of fusogenic behavior. Given the marked resistance of the hybrid liposomes to detergent-induced disruption, we next investigated whether these structural features also influenced their capacity to undergo fusion-associated membrane interactions. Membrane fusion is a highly dynamic process governed by multiple physicochemical parameters, including lipid composition, membrane charge, curvature, and bilayer organization.^45^ In particular, electrostatic attraction between cationic liposomes and negatively charged membranes is well-known to promote close membrane apposition and facilitate fusion-related processes, in some cases favoring direct membrane interaction over endocytic uptake pathways.^46,47^

Figure 6 summarizes the calorimetric and physicochemical evidence associated with the interaction of cationic hybrid liposomes with anionic conventional liposomes. Panel A shows the heat exchange profiles obtained during titration of cationic hybrid liposomes (hLP­(+)), containing DOTAP, into anionic conventional liposomes (cLP(−)) containing DOPG. These titrations produced pronounced exothermic responses, consistent with strong electrostatically driven interactions between oppositely charged bilayers. The magnitude of heat release and its progressive attenuation with successive injections are compatible with sequential membrane contact and interfacial restructuring, as expected for strongly interacting vesicular systems. For reference, the thermogram obtained for the titration of cLP­(+) into cLP(−) is presented in the Supporting Information, allowing direct comparison between the conventional cationic formulation and the hybrid systems.

**Figure 6:**
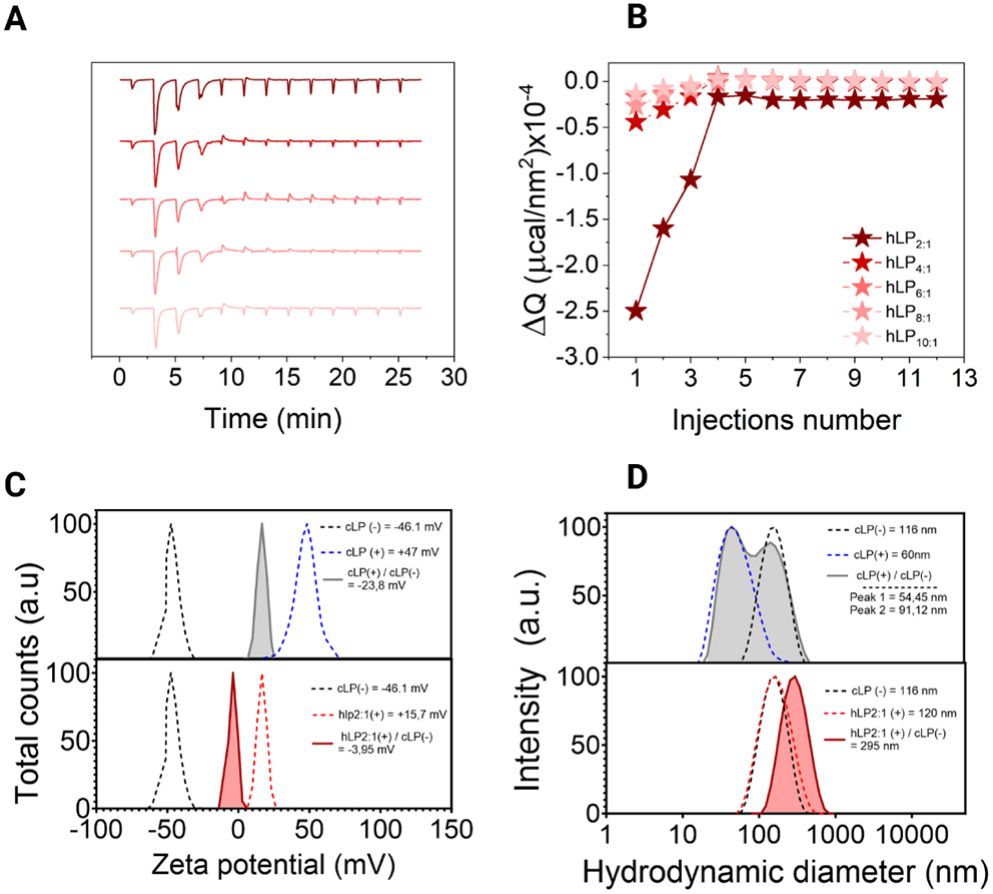
(A) Representative thermograms obtained upon titration of cationic hybrid liposomes into anionic conventional liposomes. The syringe was loaded with hLP­(+), whereas the cell contained DOPG-containing conventional acceptor vesicles, cLP(−), both at approximately 10^12^ particles/mL. Each titration comprised 13 injections of 0.3 μL, with an initial injection of 0.4 μL. (B) Heat variation per injection (Δ*Q*) as a function of injection number. (C) Zeta potential distributions of donor and acceptor vesicles, as well as postinteraction mixtures, showing surface charge redistribution after incubation. (D) Hydrodynamic diameter distributions of the individual vesicle populations and postinteraction mixtures, indicating distinct interaction outcomes for conventional and hybrid systems. All measurements were performed at 25 °C.

As shown in Figure 6B, all hLP­(+) formulations produced exothermic signals upon interaction with cLP(−), consistent with their positive zeta potentials and the electrostatic driving force expected for DOTAP-containing vesicles interacting with anionic acceptor membranes. Nevertheless, increasing the proportion of RBC membrane material in the hLP formulations enhanced the magnitude of the exothermic response. Hybrid vesicles containing higher fractions of RBC-derived components displayed more intense heat release, indicating that membrane composition strongly influences the interaction pathway. In contrast, hLPs prepared at lipid:protein ratios from 6:1 to 10:1 showed less intense calorimetric responses, with heat release approaching that observed for cLP­(+), suggesting that the exothermic signal is attenuated as RBC content decreases. This trend suggests that lower incorporation of biomembrane material is associated with weaker membrane interaction, whereas higher RBC content promotes a membrane organization more favorable to fusion-associated restructuring. One possible explanation is that increased incorporation of RBC-derived lipids and proteins generates greater lateral heterogeneity and interfacial complexity, thereby facilitating local membrane rearrangements during bilayer contact. Consistent with this view, the more exothermic thermograms observed for RBC-rich hLP formulations suggest more extensive membrane remodeling upon interaction with the anionic acceptor vesicles.

This interpretation is further supported by the zeta potential and hydrodynamic diameter measurements obtained after the titrations (Figure 6C,D). In both systems, the zeta potential distributions converged to a single intermediate peak, indicating substantial surface charge redistribution after interaction. However, the size distributions differed markedly. Titration of cLP­(+) (60 nm) into cLP(−) (116 nm) produced two size populations that remained close to those of the original vesicles, consistent with interaction and aggregation without extensive restructuring. In contrast, titration of hLP­(+) (120 nm) into cLP(−) (116 nm) resulted in a single size distribution, consistent with the formation of a new vesicular population following membrane restructuring. Although these data do not by themselves prove complete fusion, they support a fusion-associated interaction pathway that is more pronounced for the hybrid systems than for their conventional counterparts.

It is well established that ethanolamine-containing lipids, particularly phosphatidylethanolamine (PE), promote negative membrane curvature and thereby facilitate bilayer fusion by lowering the energetic barrier associated with intermediate states such as stalk formation and hemifusion.^47–49^ In addition, PE-containing membranes typically display greater flexibility, which favors the local rearrangements required for fusion. Notably, erythrocyte membranes are known to contain significant amounts of diacyl-PE species, concentrated predominantly in the inner leaflet of the plasma membrane, where PE represents one of the major phospholipid classes alongside PS and PE plasmalogen.^50^ The presence of endogenous RBC-derived PE in the hybrid liposome formulations is therefore highly likely and may contribute to the enhanced fusion-associated behavior observed for hLP­(+), serving a role directly analogous to that of DOPE in fusogenic model systems, promoting negative curvature and lowering the energetic barrier for stalk formation and hemifusion even in the absence of exogenously added auxiliary lipids. For example, Cavalcanti et al. monitored the energetics of interactions between cationic DOTAP-containing vesicles and anionic POPG-containing acceptor membranes by ITC and observed a pronounced exothermic response for fusogenic liposomes containing DOPE, whereas only minimal heat release was detected when neutral acceptor vesicles were used.^51^ This general behavior is consistent with our results, in which hLP­(+)-cLP(−) titrations produced strong exothermic signals, whereas cLP­(+)-cLP(−) interactions were dominated by weaker responses and physicochemical signatures more consistent with aggregation than extensive membrane restructuring.

Cholesterol is also known to modulate membrane fusion thermodynamics by increasing bilayer order and mechanical resistance, effects that often correlate negatively with fusion efficiency.^52,53^ In this context, the behavior of the hybrid liposomes is notable because, despite the higher cholesterol content associated with RBC membrane incorporation, the RBC-rich formulations exhibited stronger fusion-associated responses. This suggests that the effects of compositional heterogeneity and membrane protein incorporation may outweigh the fusion-suppressing influence typically attributed to cholesterol alone. Previous studies have also shown that phase separation can promote membrane fusion and lipid transfer by creating domains that favor local membrane remodeling.^54^ Based on this framework, it is plausible that RBC-derived lipids and membrane proteins introduce heterogeneous regions within the hybrid bilayer that facilitate fusion-associated membrane reorganization, even in the absence of auxiliary lipids such as DOPE. At the same time, because the present data are based on calorimetric, zeta potential, and size measurements, this mechanistic interpretation should be regarded as a working hypothesis rather than definitive proof. Nevertheless, together with the detergent-solubilization results, these findings indicate that incorporation of RBC membrane material alters not only membrane stability but also the pathway by which liposomes interact with oppositely charged membranes.

## Conclusion

The present results show that incorporation of RBC membrane material does not simply add biological complexity to phosphatidylcholine liposomes, but redefines their membrane behavior at both structural and interfacial levels. Hybridization produced bilayers with altered organization, reflected in changes in size, membrane order, thermal response, and detergent interaction profile. In particular, the increased resistance of RBC-rich formulations to TX-100 and their distinct calorimetric behavior indicate that membrane proteins and cholesterol-rich domains substantially modify the pathway by which these vesicles respond to interfacial perturbation. The interaction assays further indicate that RBC membrane incorporation alters the way cationic liposomes interact with oppositely charged membranes, promoting a more pronounced fusion-associated restructuring response than that observed for conventional liposomes. Although the present data do not directly demonstrate complete membrane fusion, they consistently support the conclusion that compositional heterogeneity introduced by RBC-derived components plays a central role in modulating membrane interaction pathways. Taken together, these findings highlight membrane hybridization as a relevant strategy for tuning the physicochemical behavior of liposomes beyond conventional lipid-only formulations. The dose-dependent effect of RBC membrane incorporation on bilayer rigidity, the enhanced resistance of RBC-rich formulations to detergent solubilization, and the altered interfacial behavior of cationic hybrid liposomes collectively indicate that biomembrane-derived components can be used to modulate both structural stability and interaction behavior in a compositionally controlled manner. More broadly, this work helps define how biomembrane-derived components reshape membrane stability and intervesicular interactions, with direct implications for the rational design of liposomal drug delivery systems, particularly in scenarios where membrane fusion, enhanced intracellular delivery, or tunable structural resistance are desirable functional outcomes.

## Supplementary Material



## Data Availability

All data generated or analyzed during this study are included in this published article.

## Notes

Transmission electron microscopy (TEM) images of hybrid liposomes; representative thermogram obtained upon titration of cationic liposome [cLP­(+)] into negative liposome [cLP(−)] and the corresponding heat variation per injection (Δ*Q*) as a function of injection number (PDF)
