# Supplementary material for: Erythrocyte Membrane Incorporation Modulates the Stability and Fusion-Associated Behavior of Biomimetic Liposomes
Source: Langmuir. 2026 Jul 30;42(31):22645–56. doi: 10.1021/acs.langmuir.6c01982 (PMC13472161; doi:10.1021/acs.langmuir.6c01982)
Supplement: Supplementary file 1 [file la6c01982_si_001.pdf]

Supporting Information: Erythrocyte Membrane Incorporation Modulates the Stability and Fusion-Associated Behavior of Biomimetic Liposomes

Lucas R. Sousa,[†,‡] Luciano V. Sá,[†,‡] Tacio G. Hayasaki,[†,¶] Leticia S. O. Freitas,[†,¶] Karin A. Riske,[§] Sebastião A. Mendanha,[*,†,‡] and Eliana M. Lima[†,¶]

†*FarmaTec – Laboratory for RD&I in Pharmaceutical Nanotechnology and Drug Delivery Systems, Samambaia Technology Park, Goiânia, GO, Brazil*

‡*Institute of Physics, Federal University of Goiás, Goiânia, GO, Brazil*

¶*School of Pharmacy, Federal University of Goiás, Goiânia, GO, Brazil*

§*Department of Biophysics, Federal University of São Paulo, São Paulo, SP, Brazil*

E-mail: sebastiao_mendanha@ufg.br

Supporting Information Figures

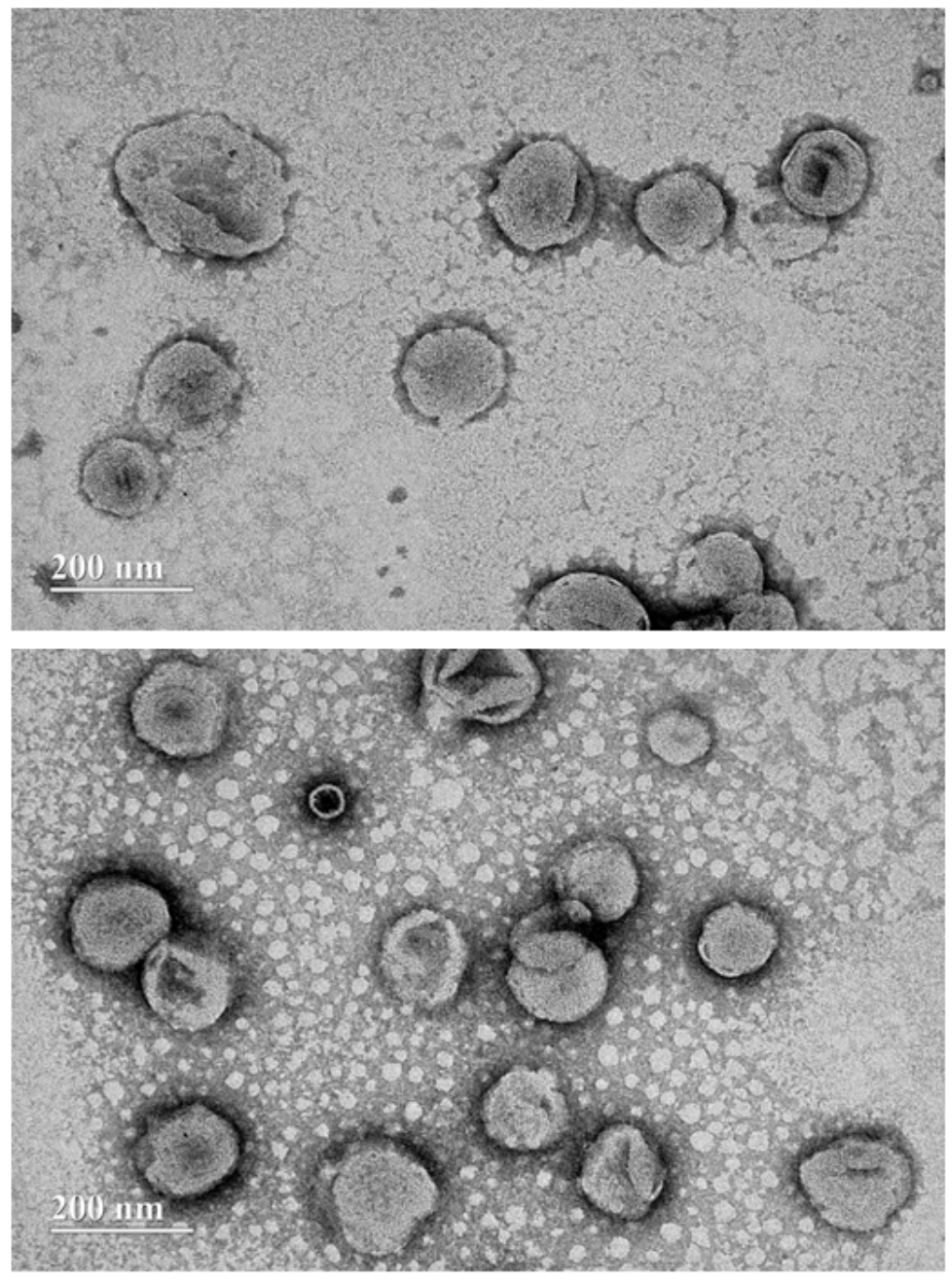

Figure S1: Transmission electron microscopy (TEM) images of hybrid liposomes.

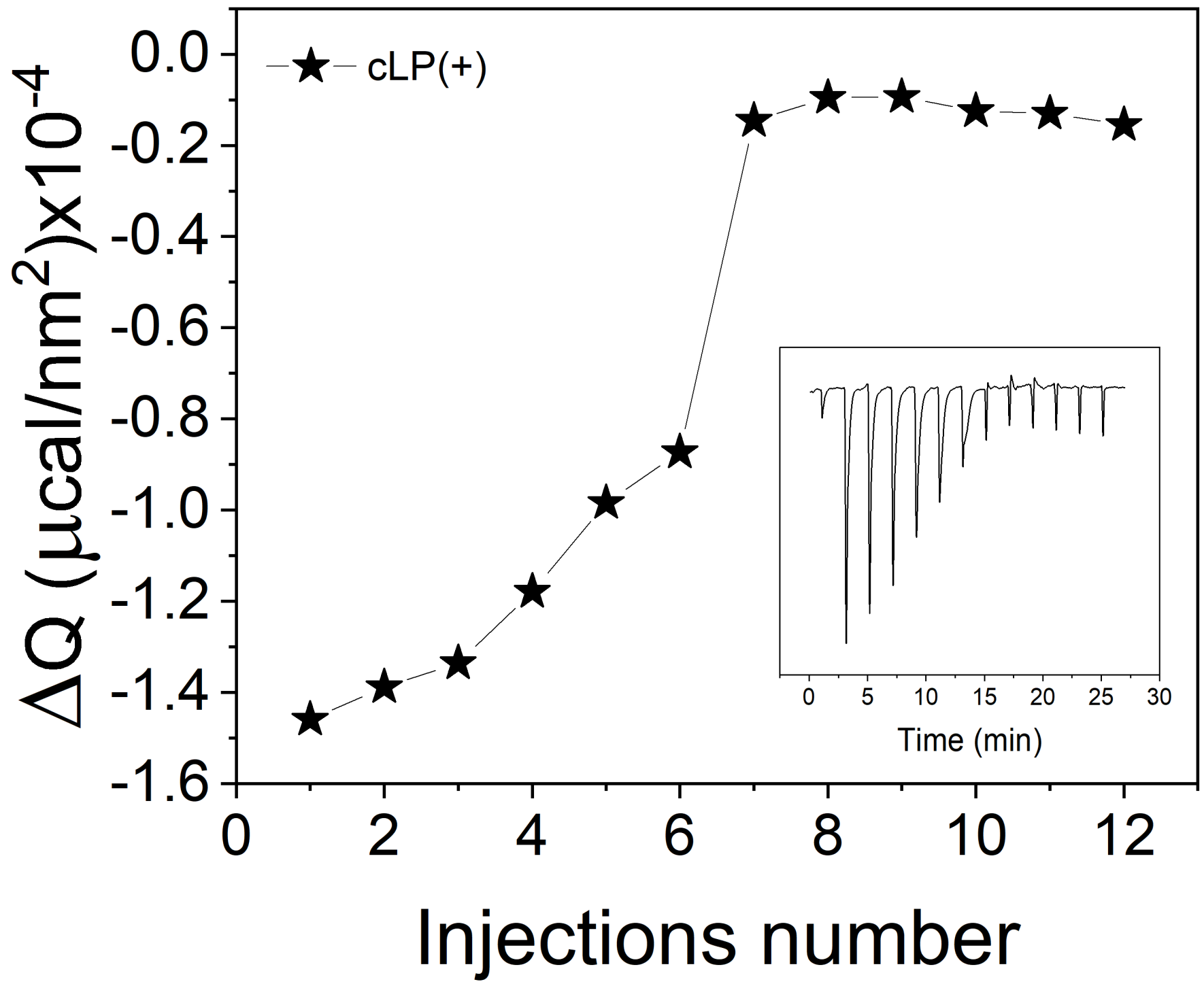

Figure S2: Representative thermogram obtained upon titration of cationic liposome [cLP(+)] into negative liposome [cLP(-)]and the corresponding heat variation per injection (ΔQ) as a function of injection number. The syringe was loaded with cLP(+) at approximately 10^{12} particles/mL. Each titration comprised 12 injections of 0.3 μL, with an initial injection of 0.4 μL. All measurements were performed at 25 °C.
